# Sociodemographic factors associated with low intake of bioavailable iron in preschoolers: National Health and Nutrition Survey 2012, Mexico

**DOI:** 10.1186/s12937-020-00567-3

**Published:** 2020-06-20

**Authors:** Yazmín Venegas-Aviles, Sonia Rodríguez-Ramírez, Eric Monterrubio-Flores, Armando García-Guerra

**Affiliations:** Centro de Investigación en Nutrición y Salud (CINYS), Instituto Nacional de Salud Pública (INSP), Av. Universidad 655 Colonia Santa María Ahuacatitlán, Cerrada Los Pinos y Caminera C.P., 62100 Cuernavaca, Mexico

**Keywords:** Iron dietary, Iron deficiency, Rural population, Child preschool, Mexico

## Abstract

**Background:**

Children < 5 years of age are at risk of developing an iron deficiency due to a low intake of bioavailable iron (FeBio). Few studies have estimated dietary FeBio in children at a national level in relation to sociodemographic characteristics. This study aimed to estimate FeBio intake and its association with sociodemographic factors among Mexican children aged 12–59 months.

**Methods:**

A cross-sectional study was carried out. Information on serum ferritin and diet was obtained from a national survey and representative sample of 1012 Mexican children aged 12–59 months. We used a 24-h recall to estimate total iron, heme and non-heme iron, vitamin C, phytates, calcium, and meat intake. We calculated FeBio intake using an algorithm. Differences in FeBio intake were analyzed by area of residence (rural/urban), country region (north, center, south), and socioeconomic status (SES), using linear regression models by age subgroups (12–23 and 24–59 months) and total population, while adjusting for study design.

**Results:**

Total iron intake was 9.2 ± 6.7 mg/d. The estimated average of total FeBio fluctuated between 0.74–0.81 mg/d, with a bioavailability of 9.15–12.03% of total iron. Children aged 12–23 months residing in rural areas consumed less FeBio than those in urban areas (β = − 0.276) (*p* < 0.05). Children aged 24–59 months with high SES consumed more FeBio (β = 0.158 mg/d) than those of a low SES (*p* < 0.05).

**Conclusions:**

FeBio is low in Mexican preschoolers. Being from a rural area and having low SES were negatively associated with FeBio intake. These results can benefit interventions seeking to improve iron status.

## Introduction

Worldwide, 47% of children under the age of five have anemia, which is mainly attributed to iron deficiency [1]. In 2012, the national prevalence of iron deficiency in Mexican preschool children was 13.6%, while prevalence of anemia was 23.3% [2].

Iron deficiency in children < 2 years of age can result in long-term cognitive deficits and psychomotor impairment [3, 4]. In preschool children, iron deficiency is associated with a lower intelligence quotient, behavioral changes, and reduced capacity for physical activity [5, 6].

Iron-deficiency anemia is due to low iron intake and more specifically, low bioavailable iron (FeBio). Inflammation, parasitic diseases, and genetic disorders are other factors known to cause anemia [1]. Iron bioavailability refers to the proportion of iron ingested, absorbed and metabolized, is essential for a number of physiological functions [7], and should be considered in diet adequacy estimates [8, 9]. The mean iron bioavailability in the United States and Canada diets is estimated at 18% for children ≥1 year [10]. However, in Mexico iron bioavailability is lower, because the Mexican traditional diet is rich in plant-based foods such as grains and legumes, an important source of phytates, which inhibit non-heme iron absorption [8, 11].

There are two forms of dietary iron: 1) heme iron, which comes from hemoglobin and myoglobin in animal source foods, with uniform absorption, and could contribute ≥40% of total absorbed iron [12]. 2) non-heme iron found in plants, animal tissues and widely present in fortified foods, and supplement compounds, its bioavailability depends on body iron reserves, iron absorption enhancers, and absorption inhibitors consumed [13, 14]. Iron absorption is estimated using stable iron isotopes [15–17]; however, this method is not feasible in population studies. For this reason, algorithms have been designed to measure its bioavailability in populations [13, 14, 18]. In Mexico, estimates found a low bioavailability of iron (2.7–6.1%), as well as an elevated intake of phytates in preschoolers’ diets [13].

Dietary patterns vary with socioeconomic characteristics, which determine iron bioavailability. In Mexico, the probability of consuming fruits, vegetables, and red meat in important quantities, is greater in individuals with a high socioeconomic status (SES) and those who reside in urban areas [19]. In Mexico, the population of northern region consumes more processed meats than in the central region [19]. The southern region follows traditional dietary patterns, with a greater consumption of plant-based foods, which influences iron intake, absorption enhancers, and absorption inhibitors [8, 19]. In Mexico, mother’s educational level was also associated with children’s healthy diets (adequate in micronutrients) [20, 21].

To our knowledge, FeBio has not been recently estimated with national data in Mexico, nor has its relationship with sociodemographic characteristics been explored. The purpose of this study is to estimate FeBio and analyze its association with sociodemographic characteristics, such as area of residence (urban/rural), region and socioeconomic status in Mexican children from 12 to 59 months of age.

## Methods

### Design and population

The present study is cross-sectional. Information was obtained from the 2012 National Health and Nutrition Survey (2012 ENSANUT, by its Spanish acronym), which is a national, probabilistic survey, with state-level, regional, and urban/rural representation. The survey was carried out between October 2011 and May 2012 [22]. The purpose of this survey was to quantify the frequency, distribution, and patterns of the Mexican population’s health and nutrition status. The 2012 ENSANUT included 50,528 households. Information on diet was obtained in a subsample of 2655 children < 5 years [23, 24]. More details on the 2012 ENSANUT can be found in other documents [22–24].

**Fig. 1:**
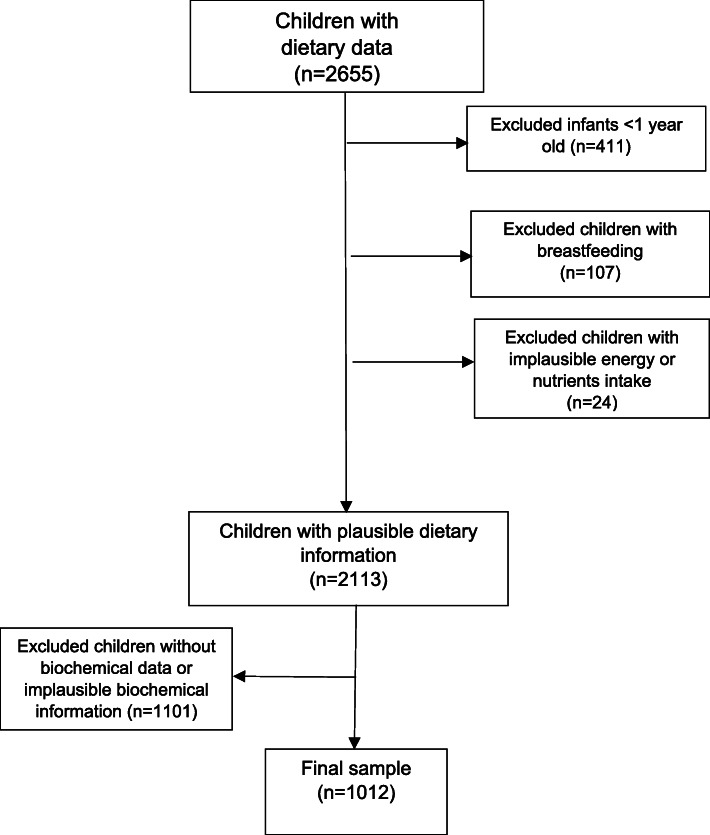
Study flowchart

This study included preschool aged children that provided a blood sample and their parents completed a 24-h dietary recall questionnaire. Of the sample with valid diet information (*n* = 2113), children whose serum ferritin concentrations (SF) and C-reactive protein (CRP) levels could not be determined were excluded (*n* = 1101) (measures described below). The final sample included 1012 children (Fig. 1).

The 2012 ENSANUT was approved by the research, biosecurity, and ethics committees at the National Institute of Public Health [Instituto Nacional de Salud Pública (INSP by its Spanish acronym)] in Cuernavaca, Morelos, Mexico. Informed consent was signed by the parents or guardians of the participating children [24].

Information on diet was obtained using a 24-h recall. The dietary information was provided by mothers or the person in charge of the child’s meals. Interviews were mostly carried out on weekday (75.1%), and a quarter took place during the weekend. A multi-step method was used in order to collect more precise information on diet [25]. This included 1) obtaining a quick or preliminary list of the foods and beverages consumed by the child, without specifying the order or time of consumption; 2) enquiring about commonly forgotten foods (predetermined list); 3) enquiring about meal time and the context in which the foods were eaten (place and activity during consumption); 4) recording detailed information about quantity and characteristics of the foods (food or beverage, consumed alone or along with other foods, ingredients used in preparation and quantities, preparation process); and finally, 5) carrying out a final revision and correcting information, if necessary.

The 24-h recall was carried out by trained personnel and standardized by researchers from INSP [24]. The 24-h recall software (multi-step methods, version 1.0, 2012; INSP) was developed and tested by INSP personnel for use in the 2012 ENSANUT.

### Estimation of nutrient intake

Intake of vitamin C, total iron, heme iron, non-heme iron, calcium, and phytates (all in mg/d) and grams of red meat, poultry, fish, and seafood were estimated using the database on nutritional values developed by INSP [26]. Additionally, in order to estimate vitamin C and phytates losses by different cooking methods, retention factors for these dietary components were used [27, 28].

### Cleaning process of diet information

A meat intake > + 3 SD was observed in 159 children and was considered implausible for the age group. For these children, we imputed mean meat consumption data by age and then recalculated the amount of heme iron, non- heme iron, and total iron consumed. A new estimate was made for the quantity of heme iron, non-heme iron, and total iron consumed. Children under 1 year of age were excluded from analyses (*n* = 411), as well as children who were partially breastfed (*n* = 107), due to the difficulty of estimating the quantity of breastmilk consumed. Values > + 3 SD or < − 3 SD of the logarithm distribution of the ratio between energy intake and energy requirement, were considered implausible and were excluded [24]. For nutrients, an upper boundary for plausible mineral intake was defined by multiplying the 99th percentile of the intake for each mineral by 1.5. When an intake value exceeded this upper boundary, we replaced it with a random value between the 95th percentile and the upper boundary value [8].

### Biochemical indicators

A venous blood sample was obtained under fasting conditions by trained personnel and were handled according to recommended laboratory procedures for storage, preservation, and processing [23, 29]. Concentrations of SF and CRP were obtained using an automatic immunoassay analyzer (Abbott diagnostics, Wiesbaden, Germany) in the INSP nutrition laboratory. Children with CRP ≥5 mg/L (*n* = 275) were excluded from analyses, considering this was due to illness and could alter SF concentrations [30, 31].

### Socioeconomic variables

Data on sex and age were obtained. As iron deficiency and its consequences are more severe in children < 2 years, children were classified in subgroups from 12 to 23 and 24–59 months [2, 4, 5]. Three geographical regions in Mexico were defined: North, Center and South. Rural areas were defined as having a population < 2500; the rest were considered as urban [22].

An index of socioeconomic status (SES) was created through the analysis of principal components, including household characteristics (number of rooms, exclusive kitchen, bathroom, use of firewood or coal as fuel, and floor material), goods and services at home (color television, microwave, washing machine, computer, motor vehicle, stereo, Internet access, cell phone, telephone line). A continuous variable was obtained and categorized in terciles (low, medium, high) [32]. Maternal education level was estimated by using the highest level of schooling, categorized as: 1) elementary school, 2) middle school, 3) high school, 4) bachelor’s degree or above.

### Estimation of bioavailable iron

The percentage of FeBio was estimated with the algorithm created by Armah et al. [14], given it had previously been used with data from nationally representative surveys in preschoolers and considered individual body iron storage. The percentage of total bioavailability was estimated along with food consumption for all meals throughout the day. Consumption of black tea was low in the study population, so this inhibiting factor was not included in the algorithm [13]. As a first step, bioavailability of non-heme iron was estimated considering absorption enhancers (vitamin C and meats), absorption inhibitors of non-heme iron (phytates and calcium), and the concentration of SF per individual using the following equation:

#### Equation I. Estimation of percentage of non-heme iron bioavailability

$$ Ln\  Bioavailability\  NHI\ \left(\%\right)=6.294-0.709\ln (SF)+0.119\ln (VC)+0.006\ln \left(M+0.1\right)-0.247\ln (Ph)-0.137\ln (ca)-0.083\ \ln (NHI) $$

Where: SF corresponded to serum ferritin (μg/L); VC to vitamin C (mg/d); M to meats (gr/d); Ph to phytates (mg/d); Ca to calcium (mg/d), and NHI to non-heme iron (mg/d).

Next, bioavailability of heme iron (HI) was estimated. According to body iron stores, two categories were defined: 1) adequate iron reserve (SF ≥12 μg/L), assuming a conservative bioavailability value of 25% for HI and 2) depleted iron reserve (SF < 12 μg/L), considering a HI bioavailability of 35% [16, 18].

As a second part of the algorithm, total iron bioavailability was estimated from the sum of the fraction of bioavailable NHI and HI with the following formula:

#### Equation II. Estimation of percentage of total iron bioavailability

$$ Total\ bioavailability=\left( Bioavailability\  NHI\ast proportion\  NHI\right)+\left( Bioavailability\  HI\ast proportion\  HI\right) $$

Where: Bioavailability NHI corresponded to the percentage of bioavailability of non-heme iron estimated in the first formula **I**; proportion NHI to the proportion from total iron in the diet (non-heme iron); bioavailability HI to the bioavailability of heme iron (conservative value of 25–35%) and proportion HI to the proportion from total iron (heme iron).

Finally, once the bioavailability of non-heme iron was obtained with the algorithm created by Armah et al. [14], FeBio was estimated in relation to dietary intake of total iron with the following formula:

#### Equation III. Estimation of bioavailable iron (in mg)

$$ Total\ FeBio=\left[ Bioavailability\  NHI\ast \left(\frac{NHI}{100}\right)\right]+\left[ Bioavailability\  HI\ast \left(\frac{HI}{100}\right)\right] $$

Where: Bioavailability NHI corresponded to the percentage of bioavailability of non-heme iron estimated from the formula **I**; NHI to non-heme dietary iron (mg/d); bioavailability HI to heme iron bioavailability (conservative value of 25–35%), and HI to heme dietary iron (mg/d).

### Statistical analysis

General characteristics of the study population are shown in proportions with confidence intervals (CI) at 95%. Using chi-square tests, statistically significant differences (*p* < 0.05) between sociodemographic factors (SES, maternal education level, area, and geographic region) by groups of age (12–23 and 24–59 months) were calculated.

Nutrient intake, concentration of serum ferritin, bioavailability of total iron and non-heme iron, heme FeBio, non-heme FeBio, and total FeBio are shown in mean ± SD, and medians. To determine mean differences in nutrient intake, statistical significance was set at *p* < 0.05, and the Bonferroni method was used to adjust for multiple comparisons [33]. To study the association between FeBio and sociodemographic factors, robust multiple linear regression modeling techniques were employed. FeBio was considered a dependent variable and SES, area, and geographic as covariates. A model was run for all children aged 12–59 months and two separate models for the groups 12–23 and 24–59 months old. The northern region, urban area, and low SES were the reference categories. We verified the normality and homogeneity of residual variance, in addition to multicollinearity between independent variables in the models. We excluded the variable mother’s education due to collinearity with SES. Analyses were performed using the logarithmic transformation of FeBio. Results were similar to the untransformed variable. To facilitate the interpretation of results, we used the untransformed variable. We used the STATA software, version 14.0, (StataCorp. 2015. Stata Statistical Software: Release 14. College Station, TX: StataCorp LP.), and adjusted by sampling design of the survey, with SVY module.

## Results

We analyzed information from 1012 children between 12 and 59 months of age who had data on diet and body iron storage indicators. Approximately half were male (48.9%), most lived in urban areas (66.2%) and in the central region and Mexico City (41.8%), 40% had a middle SES, followed by those with low SES (36.2%).

A high proportion of mothers had a middle school education level (43.1%). The sociodemographic variables (area, region, SES, and mother’s education level) were not different between the two age groups (Table 1).

**Table 1:** Sociodemographic variables in preschool children from 12 to 59 mo^a,b^

Variables		Age group	
	12–59 mo	12–23 mo	24–59 mo
	(***n*** = 1012) ***N*** = 3,773,479	(***n*** = 151) ***N*** = 472,184	(***n*** = 861) N = 3,301,294
	% (C.I. 95%)	% (C.I. 95%)	% (C.I. 95%)
**Sex**			
Male	48.9 (44.7–53.0)	47.4 (36.6–58.3)	49.1 (44.6–53.6)
Female	51.1 (46.9–55.2)	52.6 (41.6–63.3)	50.9 (46.4–55.3)
**Region, n (%)**			
North	17.0 (14.1–20.1)	15.8 (10.2–23.5)	17.0 (14.0–20.7)
Center	41.8 (37.6–46.1)	31.0 (21.4–42.6)	43.4 (38.6–48.1)
South	41.2 (37.2–45.2)	53.2 (42.6–63.5)	39.6 (35.1–44.0)
**Area, n (%)**^**a**^			
Rural	33.8 (30.3–37.5)	35.8 (26.7–46.0)	33.6 (29.6–37.7)
Urban	66.2 (62.4–69.6)	64.2 (53.9–73.2)	66.4 (62.3–70.3)
**SES, n (%)**^**b**^			
Low	36.2 (32.1–40.4)	42.2 (32.0–52.9)	35.3 (30.8–40.0)
Middle	40.0 (35.5–44.5)	41.2 (31.3–51.8)	39.8 (34.9–44.8)
High	23.8 (19.8–28.4)	16.6 (8.9–28.5)	24.9 (20.5–29.8)
**Maternal education level**^**c**^			
Elementary school	34.6 (30.2–39.0)	29.4 (20.9–39.5)	35.3 (30.6–40.2)
Middle school	43.1 (38.7–47.7)	38.7 (29.0–49.1)	43.8 (38.9–48.7)
High school	14.9 (12.0–18.2)	19.8 (12.7–29.5)	14.1 (11.1–17.8)
Bachelor’s degree or higher	7.4 (4.8–11.1)	12.1 (5.1–26.1)	6.8 (4.1–10.7)

^a^ Rural: localities with < 2500 inhabitants, urban ≥2500 inhabitants

^b^ SES: socioeconomic status, estimated by means of analyses of main components with information of the dwelling characteristic and household goods

^c^ Available information for 974 children

*Significant differences *P* < 0.05; Chi square test. Analyses adjusted by survey design

In the total sample, consumption of total iron and calcium was lower and consumption of phytates were higher in rural areas (compared to urban areas), and among children from low SES (compared with high SES) (*p* < 0.05). On the other hand, children from high SES had a higher intake of vitamin C than those from low and medium SES (*p* < 0.05), and children residing in urban areas had higher meat consumption (*p* < 0.05). No differences were observed for the intake of absorption enhancers (vitamin C and meats) or non-heme iron absorption inhibitors (phytates and calcium) between regions of residence (Table 2). Vitamin C and phytates intake were slightly higher when losses by cooking method were not considered (80 mg and 700 mg/d of vitamin C and phytates, respectively) (data not showed in table).

**Table 2:** Nutrients intake by sociodemographic factors in children from 12 to 59 mo, stratified by age^a,b^

Nutrients	Area^**c**^		Regions			SES^**d**^		
	Rural	Urban	North	Center	South	Low	Middle	High
**12 - 59 mo (*n*= 1012)**								
Total iron (mg/d)	8.11±6.2 (6.9)	9.79±6.7 (8.2)^e^	9.98±8.1 (8.6)	9.75±6.8 (6.9)	8.38±5.8 (7.3)	8.04±6.2 (6.7)^x^	9.42±7.3 (7.8)^xy^	10.68±5.8 (9.5)^y^
Non-heme iron (mg/d)	7.91±6.17 (6.8)	9.52±6.69 (8.1)^e^	9.67±8.10 (8.4)	9.47±6.81 (7.7)	8.18±5.79 (7.3)	7.85±6.22 (6.5)^x^	9.15±7.30 (7.7)^xy^	10.39±5.84 (9.2)^y^
Heme iron (mg/d)	0.19±0.35 (0.03)	0.27±0.31 (0.15)^e^	0.28±0.42 (0.18)	0.27±0.32 (0.14)	0.20±0.30 (0.05)	0.19±0.31 (0.04)^x^	0.27±0.35 (0.11)^y^	0.29±0.30 (0.19)^y^
Vitamin C (mg/d)	75.32±100.4 (58.5)	84.46±80.3 (44.6)	70.36±69.0 (51.0)	88.57±89.5 (60.2)	78.58±89.6 (50.1)	69.31±96.6 (42.9)^x^	78.57±73.6 (56.3)^x^	104.33±89.5 (77.5)^z^
Meat (g/d)	41.48±67.8 (10.2)	54.15±59.3 (35.0)^e^	51.95±73.5 (25.0)	55.33±59.4 (36.2)	43.45±61.4 (20.0)	40.99±62.4 (15.7)	52.60±64.2 (26.1)	58.72±58.2 (39.8)
Phytates (mg/d)	796.9±674.8 (699.1)	623.9±514.3 (496.0)^e^	761.6±811.9 (541.9)	613.9±450.1 (519.8)	719.6±586.9 (615.2)	769.7±642.7 (643.0)^x^	669.0±516.9 (548.2)^xy^	572.8±544.2 (438.3)^y^
Calcium (mg/d)	685.2±525.9 (627.3)	815.3±815.3 (721.9)^e^	779.5±506.8 (720.4)	815.0±479.8 (751.4)	723.5±541.9 (619.5)	657.3±502.4 (602.3)^x^	822.3±523.5 (724.5)^y^	858.7±463.9 (779.2)^y^
**12 -23 mo (*n*= 151)**								
Total iron (mg/d)	6.36±5.7 (4.9)	8.10±6.3 (7.4)	7.45±5.7 (6.6)	7.51±6.0 (6.6)	7.47±6.5 (6.5)	6.89±5.0 (6.8)	7.81±7.3 (5.6)	8.16±6.2 (7.4)
Non-heme iron (mg/d)	6.19±5.71 (4.7)	7.96±6.36 (7.4)	7.35±5.82 (5.7)	7.38±6.04 (6.2)	7.28±6.56 (6.5)	6.69±4.99 (6.5)	7.68±7.36 (5.5)	8.50±6.23 (7.4)
Heme iron (mg/d)	0.17±0.30 (0.13)	0.14±0.23 (0.23)	0.10±0.20 (0.25)	0.12±0.21 (0.16)	0.18±0.28 (0.17)	0.19±0.28 (0.25)	0.13±0.24 (0.14)	0.10±0.19 (0.26)
Vitamin C (mg/d)	80.95±153.5 (61.5)	74.37±68.9 (41.4)	81.79±89.7 (59.7)	63.28±52.3 (65.7)	83.07±124.0 (44.8)	72.41±127.6 (41.4)	72.08±75.2 (52.7)	99.29±85.5 (84.4)
Meat (g/d)	37.08±59.6 (20.7)	34.39±50.7 (9.8)	24.36±47.1 (0)	28.57±43.9 (0)	42.56±59.1 (15.7)	45.03±59.1 (21.9)	30.75±50.3 (13.5)	22.16±43.3 (0)
Phytates (mg/d)	550.8±566.2 (429.2)	449.9±546.2 (330.9)	486.9±538.3 (365.5)	340.0±252.3 (330.9)	571.0±659.7 (378.5)	636.9±719.1 (478.5)	411.4±387.1 (333.4)	287.9±289.2 (210.7)
Calcium (mg/d)	813.7±741.0 (654.2)	696.7±506.0 (608.3)	700.2±464.0 (681.8)	961.7±431.2 (632.0)	777.3±692.0 (608.3)	793.8±631.7 (654.2)	709.3±609.1 (601.8)	671.1±442.2 (420.7)
**24-59 mo (*n*= 861)**								
**Total iron (mg/d)**	8.38±6.2 (7.2)	10.02±6.7 (8.5)^e^	10.31±8.3 (9.0)	9.97±6.8 (8.0)	8.55±5.6 (7.6)	8.24±6.4 (6.7)^x^	9.65±7.2 (8.1)^x^	10.92±5.7 (9.5)^y^
**Non-heme iron (mg/d)**	8.18±6.16 (7.0)	9.73±6.68 (8.2)^e^	10.00±8.25 (8.5)	9.69±6.80 (7.8)	8.35±5.62 (7.3)	8.05±6.37 (6.5)^x^	9.36±7.23 (8.1)^xy^	10.62±5.76 (9.2)^y^
**Heme iron (mg/d)**	0.19±0.36 (0.02)	0.28±0.32 (0.17)^e^	0.30±0.43 (0.21)^x^	0.28±0.32 (0.16)^x^	0.20±0.30 (0.05)^y^	0.18±0.32 (0.03)^x^	0.29±0.35 (0.17) ^xy^	0.30±0.30 (0.23)^y^
**Vitamin C (mg/d)**	74.46±90.8 (45.7)	85.85±81.1 (58.5)^e^	68.85±66.0 (47.6)	91.16±90.8 (60.2)	77.71±82.1 (52.0)	68.78±90.5 (42.9) ^x^	79.53±72.94 (56.3)^xy^	104.81±89.7()^y^
**Meat (g/d)**	42.15±68.8 (73.2)	56.88±59.5 (50.0)^e^	55.58±74.9 (36.6)	58.07±59.5 (36.2)	43.62±61.7 (20.5)	40.30±62.9 (10.9)^x^	55.85±64.6 (35.9)^y^	62.20±58.1 (41.5)^y^
**Phytates (mg/d)**	834.5±677.1 (742)	648.0±504.6 (542.3)^e^	797.8±828.2 (556.0)	641.9±450.6 (581.0)	742.2±567.9 (641.7)	794.4.±625.8 (690.0)^x^	707.2±515.4 (608.1)^xy^	599.9±551.5.3 (459.3)^y^
**Calcium (mg/d)**	665.5±485.0 (624.1)	831.7±485.8 (737.5)^e^	790.0±509.7 (720.4)	827.6±479.2 (751.4)	713.1±510.3 (623.1)	634.0±473.3 (585.6)^x^	839.1±508.0 (740.9)^y^	876.6±461.3 (832.2)^y^

^a^Data are presented as mean ±SD (median)

^b^nalyses adjusted by survey design

^c^Rural: localities with <2500 inhabitants, urban: ≥2500 inhabitants

^d^SES: socioeconomic status, estimated by means of analyses of main components with information of the dwelling characteristic and household goods

^e^Significant difference with rural area

^x, y, z^Between categories of region and SES, values without a letter in common between columns are significant different (*p* < 0.05), adjusted by the Bonferroni method

In children aged 12–23 months, no differences were observed in the mean intake of enhancers or inhibitors of non-heme iron absorption between sociodemographic characteristics.

Children aged 24–59 months from rural areas with low SES consumed less total iron, vitamin C, and calcium and had a higher consumption of phytates, while the highest meat consumption was in children with a high SES (*p* < 0.05) (Table 2).

The mean intake of total iron by age group was 7.48 and 9.47 mg/d in children aged 12–23 months and 24–59 months, respectively (Table 3). The total estimated FeBio was 0.75 ± 0.73 mg/d in the total sample, corresponding to a bioavailability of 9.15 ± 5.36%. The main source of FeBio was non-heme iron (0.68 ± 0.70 mg/d), with a bioavailability of 8.33 ± 5.08%. In children aged 24–59 months, total intake of FeBio was 0.74 ± 0.71 mg/d, corresponding to a bioavailability of 8.74 ± 4.83%, which was similar to the estimated bioavailability in the total sample.

**Table 3:** Total and bioavailable iron intake and bioavailability in preschool children from 12 to 59 mo^a,b^

	12–59 mo (*n* = 1012)	12–23 mo (*n* = 151)	24–59 mo (*n* = 861)
	Mean ± SD (p50)	Mean ± SD (p50)	Mean ± SD (p50)
Total Iron (mg/d)	9.22 ± 6.77 (7.78)	7.48 ± 6.31 (6.62)	9.47 ± 6.77 (7.91)
Non-heme Iron (mg/d)	8.98 ± 6.72 (7.57)	7.32 ± 6.30 (6.21)	9.20 ± 6.72 (7.77)
Heme Iron (mg/d)	0.24 ± 0.33 (0.11)	0.15 ± 0.25 (0.03)	0.25 ± 0.34 (0.11)
Non-heme Iron bioavailability(%)^c^	8.33 ± 5.08 (7.01)	11.21 ± 7.46 (9.70)	7.92 ± 4.55 (6.75)
Total Iron bioavailability (%) ^d^	9.15 ± 5.36 (7.83)	12.03 ± 7.83 (10.49)	8.74 ± 4.83 (6.73)
Bioavailable non-heme Iron, (mg/d)	0.68 ± 0.70 (0.51)	0.77 ± 0.76 (0.52)	0.67 ± 0.69 (0.51)
Bioavailable heme Iron, (mg/d)^e^	0.06 ± 0.09 (0.02)	0.04 ± 0.07 (0.01)	0.06 ± 0.09 (0.03)
Total FeBio, (mg/d) ^f^	0.75 ± 0.73 (0.60)	0.81 ± 0.76 (0.59)	0.74 ± 0.71 (0.59)

^a^SD: Standar Desviation; p50: median

^b^Every estimation was adjusted by survey design

^c^Bioavailability of non-heme iron was estimated using the Armah et al. equation [14]

^d^Total iron bioavailability was estimated adding non-heme iron and heme iron bioavailabilities

^f^Bioavailable total iron was estimated adding bioavailable non-heme iron and bioavailable heme iron

^e^Bioavailable heme iron was estimated using conservative values; value = 35% if serum ferritin (SF) < 12 μg/L and value = 25% if SF ≥12 μg/L.

In children aged 12–23 months, bioavailability tended to be higher (12.03 ± 7.83%), as did FeBio (0.81 ± 0.76 mg/d) (Table 3).

Country regions were not associated with FeBio consumption in any of the three regression models. In 12 to 23-month-old children, we only found differences in the intake of FeBio by area of residence, with 0.276 mg/d less FeBio intake in rural areas versus children in urban areas (*p* < 0.05). In addition, this negative association was also observed in the total population, with 0.113 mg/d less FeBio intake in children from rural areas compared to urban areas (*p* < 0.05) (Table 4).

**Table 4:** Socioeconomic variables associated with FeBio consumption in preschool children from 12 to 59 mo^a^

Variables	12–59 mo (*n* = 1012)			12–23 mo (*n* = 151)			24–59 mo (*n* = 861)		
	β	SE	*p* value	β	SE	*p* value	β	SE	*p* value
Age (mo)	- 0.001	0.002	0.447	0.017	0.021	0.415	0.000	0.003	0.990
Regions North (Reference)									
Center	- 0.043	0.119	0.717	0.180	0.210	0.391	- 0.073	0.133	0.583
South	- 0.204	0.113	0.072	−0.009	0.149	0.950	- 0.247	0.129	0.057
Area^b^ Urban (Reference)									
Rural	- 0.113*	0.053	0.036	- 0.276*	0.123	0.026	- 0.086	0.058	0.140
SES^c^ Low (Reference)									
Middle	0.123*	0.053	0.025	0 .128	0.132	0.333	0.116	0.058	0.140
High	0.173*	0.075	0.022	0 .264	0.259	0.307	0.158*	0.079	0.047

^a^Linear regression models of FeBio consumption in children, adjusted by age, region, area and SES. Every model was adjusted by survey design

^b^Rural area: population < 2500; urban area: population ≥ 2500

^c^SES socioeconomic status

* Significant association, *P* < 0.05

In the total population, children with a middle SES consumed 0.123 mg/d and children with a high SES consumed 0.173 mg/d more FeBio than low SES (*p* < 0.05). In 24 to 59-month-old children, only differences in children with high SES were observed, with a greater intake of 0.158 mg/d FeBio in comparison to low SES (*p* < 0.05) (Table 4).

## Discussion

In this study, we found that the estimated intake of FeBio in Mexican children between 12 and 59 months of age was low (less than 1 mg/d) and was negatively associated with a low SES and residing in a rural area. We also found that dietary iron bioavailability was less than 10%. These results are due the following: 1) the majority of iron consumed in our population was non-heme, for which the bioavailability is much lower than heme iron; 2) there is a high consumption of iron absorption inhibitors, phytates and calcium, and low consumption of meat, which promotes iron absorption.

When the fraction of bioavailable heme and non-heme iron were added, a total bioavailability of 9.15 ± 5.36% was obtained, which differs from the estimated bioavailability in the United States population (15.1%) [11]. The bioavailability of iron is important to correctly estimate requirements for this nutrient. When assuming a low iron bioavailability (5.5% in children aged 1–3 years and 7.5% in children aged 4–5), estimates done with data from the Mexican National Nutrition Survey (ENN) 1999), the prevalence of iron deficiency in Mexican preschoolers was 52% [8, 34]. However, assuming a bioavailability of 18% (recommended in United States and Canada), the prevalence of iron deficiency is underestimated by 5% [8, 10]. We found that the prevalence of iron deficiency, considering the bioavailability in the present study, is 45%.

The estimated FeBio intake (0.74–0.81 mg/d) is slightly higher than previous estimates in Mexican preschool children, with data from the 1999 ENN (0.14–0.37 mg/d) [13]. Diverse factors could be contributing to the differences between estimates: 1) the instrument and methodology used for data collection were different, as in the present study a multi-step method was used, allowing for a better record of consumed foods [8, 24, 25]; 2) the algorithm applied included the concentration of SF per individual [14], whereas in 1999, three different scenarios of iron reserves were used because a ferritin measurement was not available [13]; 3) a possible change in iron intake in the past 13 years could be due to a greater consumption of fortified foods [8, 35, 36]; 4) the implementation of government programs, such as the Liconsa milk supply program (milk fortified with iron and other micronutrients), could be contributing to an improved iron status in children [36–38].

Despite increases in iron bioavailability, FeBio continues to be low, given that only 4.6% of total dietary iron is heme iron, while, in other populations, heme iron represents > 10% of total dietary iron [8, 14, 16]. It is because in 2012, 31% of households had food insecurity, which limited meat consumption within other expensive food groups [39]. There was no difference in FeBio intake between different regions in Mexico, which is largely explained by the fact that consumption of meats, vitamin C, phytates, and calcium is not different between age groups. Similar to previous studies, consumption of total iron, phytates, and vitamin C were not significantly associated with SF concentration [40].

In our analyses, there were no significant differences in FeBio intake by sex (data not shown). Children iron deficiency is reported to be attributed to factors such as muscle and blood volume increase due to rapid growth, a diet low in high available iron (heme), elevated consumption of cow’s milk, and loss of intestinal blood from parasitism, in both boys and girls [41]. Furthermore, conditions such as inflammation, vitamin deficiency, obesity, and genetic factors contribute to differences in iron absorption among individuals [12].

As our findings in the total sample suggest, living in a rural area and having a low SES are negatively associated with FeBio. In rural areas of Mexico, the traditional diet, rich in cereals and legumes, is largely consumed, representing an important source of iron and of iron absorption inhibitors [7, 8, 42]. FeBio is lower in individuals from the lowest SES terciles, which could be due to a lower mean consumption of iron absorption enhancers (meat and vitamin C) in these groups. Other studies indicated a lower consumption of fruits, vegetables (an important source of vitamin C), and red meats in individuals with a low SES, in addition to a higher consumption of energy-dense foods with low nutritional quality [20, 43].

Some strategies to increase bioavailable iron are: combination of beans or other legumes in the same meal with foods that facilitate iron absorption (citrus fruit, 100% natural juices and vegetables) [44]. Appropriate cooking methods (fermentation, sourdough preparation, soaking, and discarding soaking water) has also been effective to reduce content of phytates in whole foods [12, 45].

Our study presents some limitations. First, we performed a secondary analysis from the 2012 ENSANUT data. However, unlike clinical studies, our results can be extrapolated to the general population and provide information for health and nutrition policies. Second, the use of only a 24-h recall and a possible overestimation of the prevalence of iron deficiency. Nonetheless, the 24-h multi-step recall method minimizes omission of forgotten foods and improves diet estimates [25]. Third, not adjusting the calcium and iron intake for losses for cooking methods. However, we consider that the lack of adjustment for losses of these two minerals does not change the direction of the results, since the highest cooking losses for calcium and iron are found in vegetables ~ 24% of the total losses, and a high proportion of Mexican children < 48 months do not consume vegetables (80%) [43]. For iron, losses by cooking oscillate between 5 and 10% in the different food [27, 46]. Fourth, information on dietary supplements was not collected in this sample of children, which would have been useful to have a better estimate of the iron intake.

The strengths of the study include the use of an algorithm developed with national data, proved useful for estimating iron bioavailability in population groups [11]. Another strength is the estimation of vitamin C and phytates intakes by adjusting for losses in relation to the different cooking methods used. This adjustment provided a better estimate of the total bioavailable iron [28]. Furthermore, to the best of our knowledge, few studies have estimated FeBio in preschool children’s overall diet at a national level, in a representative manner.

## Conclusions

In conclusion, the estimated FeBio in Mexican children was low. Rural areas and low SES were negatively associated with FeBio intake. From a public health standpoint, identifying dietary factors that hinder or promote iron bioavailability in groups with certain sociodemographic characteristics will allow for the design of targeted interventions to improve consumption of bioavailable iron. In light of the present findings, future studies should review iron intake recommendations in the Mexican population.

## Data Availability

The dataset used for analysis is available from the corresponding author on reasonable request.

## Abbreviations

CRP: C-reactive protein
ENN: National Nutrition Survey
ENSANUT: National Health and Nutrition Survey
FeBio: Bioavailable iron
HI: Heme iron
INSP: National Institute of Public Health (Instituto Nacional de Salud Publica)
M: Meat
NHI: Non-heme iron
Ph: Phytates
SES: Socioeconomic status
SF: Serum ferritin
VC: Vitamin C
